# Evaluation of oral anticoagulants in atrial fibrillation patients over 80 years of age with nonsevere frailty

**DOI:** 10.1002/joa3.12231

**Published:** 2019-09-06

**Authors:** Masaya Shinohara, Ryou Wada, Shintaro Yao, Kensuke Yano, Katsuya Akitsu, Hideki Koike, Toshio Kinoshita, Hitomi Yuzawa, Takeya Suzuki, Tadashi Fujino, Takanori Ikeda

**Affiliations:** ^1^ Department of Cardiovascular Medicine Toho University Graduate School of Medicine Tokyo Japan

**Keywords:** bleeding, direct oral anticoagulants, elderly, frail, warfarin

## Abstract

**Background:**

The safety and efficacy of an oral anticoagulant (OAC) treatment and the difference between direct OACs (DOACs) and warfarin in nonsevere frail elderly patients with AF are unclear.

**Methods:**

This was a retrospective and observational study of 354 patients over 80 years of age with nonsevere frailty who were diagnosed with AF and treated with OACs. Nonsevere frailty was defined as a clinical frailty scale score of <7. Bleeding and thromboembolic events during the OAC treatment were followed up.

**Results:**

Of 354 patients enrolled, 273 (77.1%) received DOACs and 81 (22.9%) received warfarin. Of 273 patients receiving DOACs, there were 210 (76.9%) prescribed with appropriate doses of DOACs. Of 81 warfarin‐treated patients, 53 (65.4%) were prescribed an appropriate dose of warfarin. During a follow‐up of 33.1 (14.0‐51.0) months, 15 patients (1.5/100 person‐years) had bleeding events and 10 (1.0/100 person‐years) had thromboembolic events while on an OAC treatment. The incidence ratio of bleeding events in patients receiving DOACs was lower than that in those receiving warfarin (1.0/100 person‐years vs 2.9/100 person‐years, hazard ratio [HR]: 0.26, 95% confidence interval [CI]: 0.07‐0.91, *P* = .036). There was no significant difference in the incidence of thromboembolic events between the DOAC and warfarin treatment groups (0.88/100 person‐years vs 1.4/100 person‐years, HR: 0.63, 95% CI: 0.16‐2.57, *P* = .52).

**Conclusions:**

OACs are substantially safe and effective for preventing thromboembolic events in nonsevere frail patients over 80 years of age. Particularly, DOACs can be used more safely than warfarin.

## Graphical abstract

Oral anticoagulants (OACs) are substantially safe and effective for preventing thromboembolic events in non‐severe frail patients over 80 years of age. Particularly, direct OACs can be used more safely than warfarin.

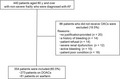

## 1 INTRODUCTION

Atrial fibrillation (AF) is the most common type of arrhythmia in clinical practice. It is estimated that AF is associated with approximately 30% of ischemic strokes in patients over 80 years of age.^1^ The elderly population is growing worldwide, and is expected to result in a more than four‐fold burden of AF among patients over 80 years of age by the year 2050, representing more than half of all cases of AF.^2^

The evaluation of frailty is important to help assess the suitability of an oral anticoagulant (OAC) treatment for preventing thromboembolic events in AF patients. Frailty has been found to be associated with poor clinical outcomes related to medical management.^3^ Following this, in severe frail elderly patients with AF, it may be appropriate to use no anticoagulation to avoid bleeding events.^4^ A previous study reported that patients classified as being nonsevere frail patients were 3.5 times more likely to receive OACs than the severe frail patients.^5^ However, there are limited data about the safety and efficacy of an OAC treatment in octogenarians whose frailty is not so severe.

The objective of this study was to evaluate the safety and efficacy of an OAC treatment and the difference between direct OACs (DOACs) and warfarin for the management of AF among nonsevere frail patients over 80 years of age.

## 2 METHODS

### 2.1 Study population

This was a retrospective and observational study of patients over 80 years of age with nonsevere frailty who were diagnosed with AF under regular outpatient care or under admission and were newly treated with the DOACs (dabigatran, rivaroxaban, apixaban or edoxaban) or warfarin to prevent thromboembolic events between January 2011 and August 2017. All patients were treated at our institution. The patient observation began at the start of the use of the OACs. The start date of the OACs was considered to be the date the prescription was dispensed. Patients were categorized into DOAC or warfarin groups based on their administered OACs. We also included the patients treated with antiplatelet drugs in combination with OACs.

### 2.2 Assessment of frailty

Frailty was assessed with the Clinical Frailty Scale (CFS) of the Canadian Study on Health & Aging, which has been verified as a useful rapid assessment tool of frailty.^6^, ^7^ The CFS is a measure of frailty based on a clinical judgement that takes into account cognition, mobility, function, and co‐morbidities, with scores ranging from 1 (very fit) to 9 (terminally ill).^8^ Each patient was attributed a CFS score by each physician when AF was diagnosed. Nonsevere frailty was defined as a CFS score of <7 in the present study.

### 2.3 OAC treatment regimens

Decisions regarding the choice of the OACs, whether the four DOACs or warfarin, was left to the discretion of each physician. We decided the dosages of the DOACs based on the approved Japanese recommendations, either dabigatran 150 mg twice daily (110 mg twice daily in patients with a creatinine clearance [CrCl] of 30‐49 mL/minute [min] or over 70 years of age), rivaroxaban 15 mg once daily (10 mg once daily in patients with a CrCl of 15‐49 mL/min), apixaban 5 mg twice daily (2.5 mg twice daily in patients with at least two of the following criteria: age ≥ 80 years, body weight [BW] ≤60 kg, or serum creatinine ≥ 1.5 mg/dL), or edoxaban 60 mg once daily (30 mg once daily in patients with a BW ≤60 kg or a CrCl of 15‐49 mL/min). According to the package, there is no way that a low dose of dabigatran prescribed for patients over 80 years of age is regarded as an inappropriately low dose. So, in the present study, we defined “an inappropriately low dose of dabigatran” as a dabigatran dose of < 220 mg/d (for example, 75 mg twice daily) without an indication for preventing thromboembolic events. The warfarin dose was adjusted to a target prothrombin time‐international normalized ratio (PT‐INR) of 1.6‐2.6 in accordance with the Japanese guidelines for AF treatment.^9^ The time in a therapeutic INR range (TTR) was calculated by using the method of Rosendaal et al^10^ The appropriate dose of warfarin was defined as a TTR with a PT‐INR value between 1.6 and 2.6 for over more than 65% of the entire treatment period.

### 2.4 Study outcomes

The primary safety outcome of the study was represented by the incidence rate of bleeding events composed of major bleeding (MB) and clinically relevant non‐major bleeding (CRNMB). The primary efficacy outcome included the incidence of thromboembolic events composed of ischemic strokes and systemic embolisms (SEs). Because of the retrospective characteristic of this study, the incidence ratio of bleeding or thromboembolic events did not directly reflect the safety or efficacy of each OAC alone. To monitor the adverse effects due to OACs, interviewing and examining the patients or obtaining usual blood tests, were performed every 1‐3 months during the OAC treatment. Additional testing was performed as necessary when a bleeding or thromboembolic event was suspected. MB was defined as bleeding requiring hospitalization, bleeding requiring a transfusion of at least 2 units of packed red cells, or bleeding occurring at a critical site during the use of OACs. CRNMB was defined as bleeding not meeting the criteria for major bleeding, but requiring medical intervention, unscheduled contact with a physician, or temporary cessation of the OAC treatment. Ischemic strokes were defined as a loss of neurological function of a sudden onset lasting ≥24 hours. SEs were defined as thromboembolisms outside the brain. Patients were followed until their first bleeding or thromboembolic event, discontinuation of the treatment, a treatment switch to a different OAC, patient death, or the end of the study period (August, 2018).

### 2.5 Statistical analysis

Data analyses were performed using EZR on R‐commander version 1.24 software (Saitama Medical Center, Jichi Medical University). All continuous variables were tested for the normality of the distribution using the Kolmogorov‐Smirnov test. Continuous variables with a normal distribution were described as the mean ± standard deviation (SD), continuous variables with a skewed distribution were described as the median (quartile: 25%‐75%), and categorical variables were described as frequencies and percentages. Comparisons between groups were analyzed by univariate logistic analysis (Fisher's exact test, Unpaired *t* test, or Mann‐Whitney test) and multivariate analysis using a logistic regression analysis. The incidence ratio was calculated using the person‐year method (events per 100 person‐years). The relationship of the OAC treatment and the incidence ratio of bleeding or thromboembolic events was analyzed using the Kaplan‐Meier method, and the curves were compared using a log‐rank test. A multivariate analysis using a Cox proportional hazard model was constructed to assess the development of bleeding and thromboembolic events. These models were adjusted by the age, gender, HAS‐BLED score, BW, CrCl, usage of antiplatelet drugs, and dosages of OACs for bleeding events; and adjusted by the age, hypertension, diabetes mellitus, past history of an ischemic stroke, and CHADS_2_ score for thromboembolic events. In all tests, a *P*‐value of .05 was considered as the cut‐off for statistical significance.

### 2.6 Ethical consideration

This study was performed in accordance with the Code of Federal Regulations and the Declaration of Helsinki. The present study was approved by the Toho University Omori Medical Center Ethical Committee (number: M17139), and informed consent was obtained from each patient before the study.

## 3 RESULTS

### 3.1 Patient characteristics

**Figure 1:**
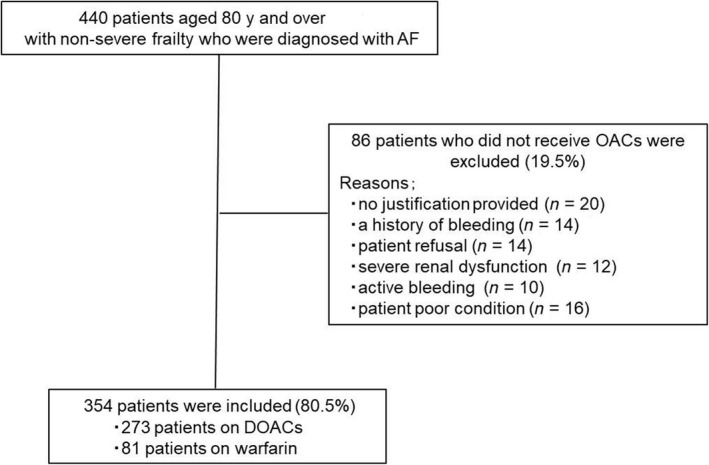
Flow chart of the study. Three hundred fifty‐four consecutive nonsevere frail octogenarians who initiated OACs were included in the present study. Abbreviations: AF, atrial fibrillation; OACs, oral anticoagulants; DOACs, direct oral anticoagulants

Among 440 consecutive patients aged 80 years and over with nonsevere frailty who were diagnosed with AF, we retrospectively analyzed 354 patients who were treated with DOACs (dabigatran, rivaroxaban, apixaban or edoxaban) or warfarin treatment at our institution between January 2011 and August 2017. The reasons why 86 patients were not treated with OACs were no justification provided (23.3%), a history of bleeding (16.3%), patient refusal (16.3%), severe renal dysfunction (14.0%), active bleeding (11.6%), or a poor patient condition (18.6%). The study flow chart is shown in Figure 1. The mean age was 83.8 ± 3.6 years, and 48.0% were male. The mean BW was 52.1 ± 11.0 kg and mean body mass index (BMI) 21.6 ± 3.9 kg/m^2^. The CFS was 1, 2, 3, 4, 5, and 6 in 2, 7, 104, 146, 70, and 25 of 354 patients, respectively. Eighty patients received antiplatelet drugs in combination with OACs. Among them, 16 patients received dual antiplatelet therapy (DAPT). The reason why the 80 patients were treated with antiplatelet therapy was coronary artery disease (CAD) including post percutaneous coronary intervention (PCI), peripheral arterial disease, and prevention of ischemic strokes. The mean follow‐up period was 33.1 (14.0‐51.0) months. The baseline clinical details are shown in Table 1.

**Table 1:** Baseline characteristics

All patients (n = 354**)**	
Male, number (%)	170 (48.0%)
Age (y)	83.8 ± 3.6
Body mass index (kg/m^2^)	21.6 ± 3.9
Body weight (kg)	52.1 ± 11.0
Hypertension, number (%)	273 (77.1%)
Diabetes mellitus, number (%)	78 (22.0%)
Congestive heart failure, number (%)	117 (33.1%)
Ischemic stroke, number (%)	71 (20.0%)
Coronary artery disease, number (%)	48 (13.6%)
PCI using stents (+)	36 (10.2%)
Smoking, number (%)	150 (42.4%)
Paroxysmal AF, number (%)	197 (55.6%)
Dementia (%)	54 (15.3%)
COPD (%)	17 (4.9%)
History of bleeding (%)	20 (5.6%)
CHADS_2_ score	2.7 ± 1.1
CHA_2_DS_2_‐VASc score	5.3 ± 1.3
Clinical Frailty Scale	4.0 ± 0.9
HAS‐BLED score	2.2 ± 0.8
Serum creatinine (mg/dl)	0.9 ± 0.3
eGFR (mL/min/1.73 m^2^)	55.3 ± 17.4
Creatinine clearance (mL/minute)	44.7 ± 16.1
Hemoglobin (ng/dL)	12.3 ± 1.8
Use of warfarin, number (%)	81 (22.9%)
Antiplatelet therapy, number (%)	80 (22.6%)
Aspirin	42 (11.9%)
ADP receptor inhibitors	19 (5.4%)
PDE3 inhibitors	12 (3.4%)
Others	24 (6.8%)
Treatment follow up (in mo)	33.1 (14‐51)

Note

Data are expressed as the mean ± SD, median (25%‐75%), or number (%).

Abbreviations: ADP, adenosine diphosphate; AF, atrial fibrillation; COPD, Chronic Obstructive Pulmonary Disease; eGFR, estimated glomerular filtration rate; PCI, percutaneous coronary intervention; PDE3, phosphodiesterase enzyme 3.

### 3.2 OAC prescription

Overall, 273 (77.1%) received DOACs (dabigatran, 64 patients; ribaroxaban, 81 patients; apixaban, 100 patients; edoxaban, 28 patients), and 81 (22.9%) received warfarin. Among the 273 patients receiving DOACs, a total of 210 patients (76.9%) were treated with appropriate doses of DOACs (dabigatran, 47 patients; ribaroxaban, 61 patients; apixaban, 77 patients; and edoxaban, 25 patients), while in the remaining patients, 42 (15.4%) received inappropriately low doses of DOACs (dabigatran, 10 patients; ribaroxaban, 13 patients; apixaban, 17 patients; and edoxaban, 2 patients) and 21 (7.7%) inappropriately high doses of DOACs (dabigatran, 7 patients; ribaroxaban, 7 patients; apixaban, 6 patients; and edoxaban, 1 patient). The inappropriate doses of DOACs were prescribed based on clinical judgement. On the other hand, among 81 patients prescribed warfarin, 53 (65.4%) were prescribed an appropriate dose of warfarin. The mean TTR in the patients with an appropriate dose of warfarin was 69.8%. An inappropriate dose of warfarin was prescribed more often than inappropriate doses of DOACs (34.6% vs 23.1%, odds ratio [OR]: 1.92, 95% confidence interval [CI]: 1.12‐3.29, *P* = .043). Table 2 shows the clinical details of each subgroup, that is, according to the prescribed OACs and dosages.

**Table 2:** Clinical details according to the prescribed OACs and dosages

	Dabigatran, appropriate dose	Dabigatran, inappropriate dose		*P* value
		Low dose	High dose	
Male	25 (53.2%)	2 (20.0%)	2 (28.6%)	.086
Age (y)	82.9 ± 2.9	85.6 ± 4.9	82.8 ± 3.6	.099
BW (kg)	50.7 ± 11.4	46.2 ± 12.5	57.0 ± 8.5	.31
CrCl (mL/minute)	45.3 ± 13.5	39.5 ± 14.0	56.8 ± 26.9	.16
CHADS_2 _score	2.7 ± 1.2	2.6 ± 0.9	2.8 ± 1.5	.99
Clinical frailty scale	3.9 ± 0.9	4.3 ± 0.9	3.8 ± 1.3	.14
HAS‐BLED score	2.2 ± 0.8	2.1 ± 0.6	2.3±0.3	.96

	Rivaroxaban, appropriate dose	Rivaroxaban, inappropriate dose		*P* value
		Low dose	High dose	
Male	28 (45.9%)	4 (30.8%)	5 (71.4%)	.097
Age (y)	84.1 ± 3.8	84.7 ± 3.5	84.5 ± 4.8	.91
BW (kg)	51.6 ± 10.9	54.7 ± 11.0	42.7 ± 6.4	.077
CrCl (mL/minute)	46.5 ± 15.7	50.1 ± 10.1	45.5 ± 10.6	.7
CHADS_2 _score	2.6 ± 1.2	2.4 ± 0.9	2.5 ± 0.8	.8
Clinical frailty scale	4.0 ± 0.9	4.2 ± 0.9	4.2 ± 1.5	.64
HAS‐BLED score	2.1 ± 0.7	1.9 ± 0.5	2.3 ± 0.8	.44

	Apixaban, appropriate dose	Apixaban, inappropriate dose		*P* value
		Low dose	High dose	
Male	32 (41.6%)	13 (76.5%)	3 (50.0%)	<.01
Age (y)	83.9 ± 3.7	82.5 ± 2.6	81.6 ± 1.1	.14
BW (kg)	51.7 ± 11.3	64.1 ± 8.0	52.4 ± 12.7	<.01
CrCl (mL/minute)	45.0 ± 17.9	53.9 ± 11.6	52.1 ± 12.5	.12
CHADS_2 _score	2.7 ± 1.0	3.1 ± 1.3	1.8 ± 0.8	.042
Clinical frailty scale	4.0 ± 0.9	3.9 ± 0.8	3.6 ± 0.9	.48
HAS‐BLED score	2.2 ± 0.8	2.4 ± 0.9	1.5 ± 0.5	.062

	Edoxaban, appropriate dose	Edoxaban, inappropriate dose		*P* value
		Low dose	High dose	
Male	12 (48.0%)	1 (33.3%)	0 (0%)	1
Age (y)	83.7 ± 3.5	86.7 ± 5.9	88.0 ± NA	.23
BW (kg)	53.0 ± 10.0	65.1 ± 5.0	52.0 ± NA	.16
CrCl (mL/minute)	48.6 ± 16.3	60.6 ± 3.6	34.8 ± NA	.32
CHADS_2 _score	2.7 ± 1.3	2.3 ± 1.2	5.0 ± NA	.16
Clinical frailty scale	4.4 ± 0.9	4.3 ± 0.6	5.0 ± NA	.76
HAS‐BLED score	2.2 ± 1.1	1.7 ± 0.6	3.0 ± NA	.51

	Warfarin, appropriate dose	Warfarin, inappropriate dose	*P* value
Male	26 (49.1%)	15 (53.6%)	.82
Age (y)	84.9 ± 3.9	83.6 ± 3.7	.16
BW (kg)	49.9 ± 9.3	53.5 ± 10.5	.11
CrCl (mL/minute)	36.2 ± 16.9	39.6 ± 12.6	.36
CHADS_2 _score	3.0 ± 1.3	2.6 ± 1.0	.18
Clinical frailty scale	4.2 ± 1.0	3.6 ± 0.9	.01
HAS‐BLED score	2.6 ± 0.9	2.2 ± 0.7	.059

Note

Data are expressed as the mean ± SD, or number (%).

Abbreviations: BW, body weight; CrCl, creatinine clearance.

*P* < .05 was considered as significant.

### 3.3 Primary safety outcome

During the follow‐up of 33.1 (14.0‐51.0) months, 15 patients had bleeding events while on OAC treatment (1.5/100 person‐years). Of those patients, 13 patients had MB, and 2 had CRNMB. The 15 bleeding events involved one with hemoptysis, one with hepatic bleeding, and thirteen with gastrointestinal bleeding events. No patients in the dabigatran group (0/100 person‐years), 5 in the rivaroxaban group (2.4/100 person‐years), 1 in the apixaban group (0.5/100 person‐years), 1 in the edoxaban group (3.4/100 person‐years) and 8 in the warfarin (2.9/100 person‐years) group experienced bleeding events. The incidence ratio of bleeding events did not differ significantly among each of the four DOACs (*P* = .079). All of the bleeding patients treated with DOACs had a CFS score of ≥4 (7/7, 100%). Among the 8 patients that experienced bleeding events while receiving warfarin, 5 had INR values over 3.0 at the time of the bleeding event or during the preceding 7 days. One patient prescribed warfarin died from massive hematemesis due to a peptic ulcer. The incidence of bleeding events in each OAC subgroup, that is, according to the prescribed OACs and dosages is presented in Table 3.

**Table 3:** Incidence of bleeding events according to the prescribed OACs and dosages

	MB, n/N (%)	CRNMB, n/N (%)
Dabigatran, appropriate dose	0/47 (0)	0/47 (0)
Dabigatran, inappropriate dose		
Low dose	0/10 (0)	0/10 (0)
High dose	0/7 (0)	0/7 (0)
Rivaroxaban, Appropriate dose	3/61 (1.9)	1/61 (0.6)
Rivaroxaban, inappropriate dose		
Low dose	1/13 (3.0)	0/13 (0)
High dose	0/7 (0)	0/7 (0)
Apixaban, Appropriate dose	1/77 (0.6)	0/77 (0)
Apixaban, inappropriate dose		
Low dose	0/17 (0)	0/17 (0)
High dose	0/6 (0)	0/6 (0)
Edoxaban, Appropriate dose	0/25 (0)	0/25 (0)
Edoxaban, inappropriate dose		
Low dose	1/2 (48.1)	0/2 (0)
High dose	0/1 (0)	0/1 (0)
Warfarin, appropriate dose	3/53 (1.7)	1/53 (0.6)
Warfarin, in appropriate dose	4/28 (4.2)	0/28 (0)

Note

Data are expressed as the number (incidence rates). Incidence rates are events per 100 person‐years.

Abbreviations: MB, major bleeding; CRNMB, clinically relevant non‐major bleeding.

### 3.4 Primary efficacy outcome

During the follow‐up period, thromboembolic events occurred in 10 patients (1.0/100 person‐years). Of those patients, 8 had an ischemic stroke, and 2 a SE. Three patients in the dabigatran group (1.2/100 person‐years), 1 in the rivaroxaban group (0.5/100 person‐years), 1 in the apixaban group (0.5/100 person‐years), 1 in the edoxaban group (3.4/100 person‐years) and 4 in the warfarin group (1.4/100 person‐years) experienced thromboembolic events. The incidence ratio of thromboembolic events did not differ significantly among each of the four DOACs (*P* = .053). Table 4 shows the incidence of thromboembolic events in each OAC subgroup, that is, according to the prescribed OACs and dosages.

**Table 4:** Incidence of thromboembolic events according to the prescribed OACs and dosages

	IS, n/N (%)	SE, n/N (%)
Dabigatran, appropriate dose	2/47 (1.1)	1/47 (0.6)
Dabigatran, inappropriate dose		
Low dose	0/10 (0)	0/10 (0)
High dose	0/7 (0)	0/7 (0)
Rivaroxaban, appropriate dose	0/61 (0)	0/61 (0)
Rivaroxaban, inappropriate dose		
Low dose	1/13 (2.8)	0/13 (0)
High dose	0/7 (0)	0/7 (0)
Apixaban, appropriate dose	1/77 (0.6)	0/77 (0)
Apixaban, inappropriate dose		
Low dose	0/17 (0)	0/17 (0)
High dose	0/6 (0)	0/6 (0)
Edoxaban, appropriate dose	1/25 (2.0)	0/25 (0)
Edoxaban, inappropriate dose		
Low dose	0/2 (0)	0/2 (0)
High dose	0/1 (0)	0/1 (0)
Warfarin, appropriate dose	2/53 (1.2)	0/53 (0)
Warfarin, in appropriate dose	1/28 (1.1)	1/28 (1.1)

Note

Data are expressed as the number (incidence rates). Incidence rates are events per 100 person‐years.

Abbreviations: IS, ischemic stroke; SE, systemic embolism.

### 3.5 Comparison of the safety and efficacy outcome between DOACs and warfarin

**Figure 2:**
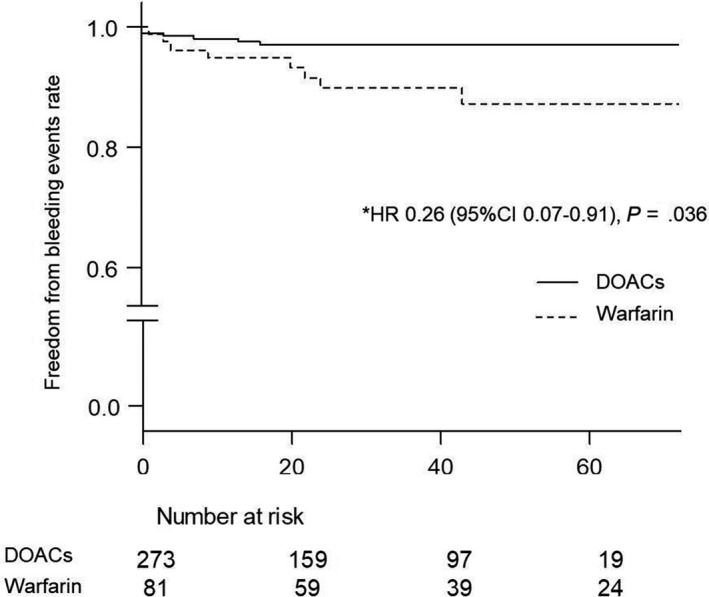
Kaplan‐Meier curves regarding the bleeding events during the follow‐up period. This figure shows the comparison of the bleeding events between DOAC and warfarin treatment. The normal line represents DOACs. The dotted line represents warfarin. Abbreviations: HR, hazard ratio; CI, confidence interval; DOACs, direct oral anticoagulants. *Adjusted by the age, gender, HAS‐BLED score, BW, CrCl, the usage of antiplatelet drugs, and the dosages of OACs. The rate differed significantly between the two groups

**Figure 3:**
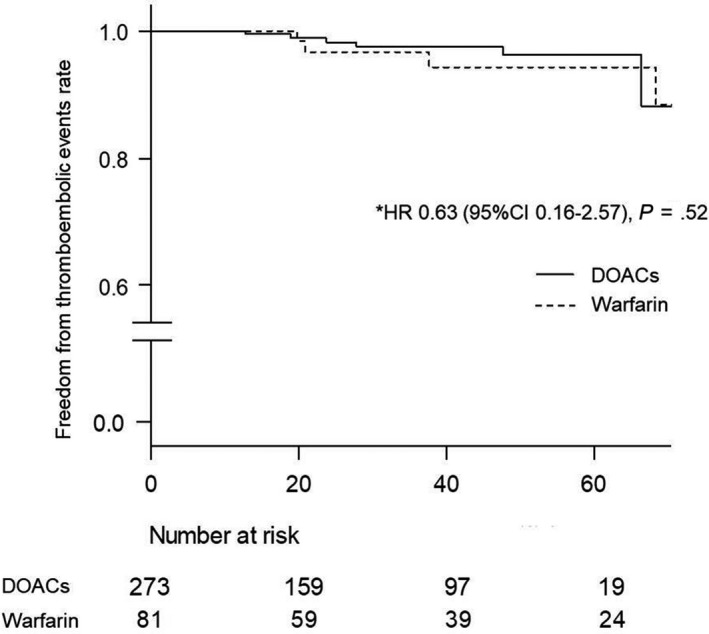
Kaplan‐Meier curves regarding the thromboembolic events during the follow‐up period. This figure shows the comparison of the thromboembolic events between DOAC and warfarin treatment. The normal line represents DOACs. The dotted line represents warfarin. HR indicates hazard ratio; CI, confidence interval; DOACs, direct oral anticoagulants. *Adjusted by the age, hypertension, diabetes mellitus, past history of an ischemic stroke, and CHADS_2_ score. The rate did not differ significantly between the two groups

Figure 2 shows the results of the Kaplan‐Meier curves regarding bleeding events between the DOAC and warfarin treatment after the OAC administration. The occurrence ratio of bleeding events in patients receiving DOACs was lower than that in those receiving warfarin (1.0/100 person‐years person‐year vs 2.9/100 person‐years, hazard ratio [HR]: 0.26, 95% CI: 0.07‐0.91, *P* = .036). On the other hand, the incidence of thromboembolic events did not differ between the DOAC and warfarin treatment groups (0.88/100 person‐years vs 1.4/100 person‐years, HR: 0.63, 95% CI: 0.16‐2.57, *P* = .52) (Figure 3).

### 3.6 Comparison of the outcome according to the quality of the OAC treatment

#### 3.6.1 DOAC treatment

The patients receiving DOACs were divided into three groups, appropriate doses of DOACs (n = 210), inappropriately low doses of DOACs (n = 42), and inappropriately high doses of DOACs (n = 21). The BW was significantly higher in patients receiving inappropriately low doses of DOACs compared to those receiving appropriate doses of DOACs or inappropriately high doses of DOACs (57.6 kg vs 51.6 kg vs 49.9 kg, *P* < .01). The incidence of bleeding events (0.95/100 person‐years vs 1.9/100 person‐years vs 0/100 person‐years, log‐rank test, *P* = .57) and thromboembolic events (0.95/100 person‐years vs 0.95/100 person‐years vs 0/100 person‐years, log‐rank test, *P* = .77) did not differ among the three treatment groups, respectively.

#### 3.6.2 Warfarin treatment

The patients receiving warfarin were divided into two groups, an appropriate dose of warfarin group (n = 53) and an inappropriate dose of warfarin group (n = 28). There was no difference in the baseline clinical characteristics between the two groups. Compared to an inappropriate dose of warfarin, an appropriate dose of warfarin had slightly lower bleeding events (2.2/100 person‐years vs 4.2/100 person‐years, HR: 0.19, 95% CI: 0.035‐1.00, *P* = .05) and similar thromboembolic events (1.1/100 person‐years vs 2.1/100 person‐years, HR: 0.18, 95% CI: 0.023‐1.45, *P* = .11).

## 4 DISCUSSION

### 4.1 Main findings

Our data suggested that OACs are substantially safe and effective for reducing the risk of AF‐associated thromboembolic events in nonsevere frail patients over 80 years of age. Particularly, DOACs can be used more safely than warfarin.

### 4.2 Assessment of the safety and efficacy outcome

During the follow‐up period, there were few bleeding events and rare thromboembolic events. The present study had a non‐inferior safety and efficacy outcome while on the OAC treatment compared to the real‐world studies that included patients older than 65 years.^11^, ^12^ Our data revealed that the OAC treatment was safe and effective for the management of AF in octogenarians if their frailty was not so severe.

### 4.3 Comparison of the safety and efficacy outcome between DOACs and warfarin

While it is likely that the DOACs will eventually replace warfarin for the management of AF in clinical practice, the role of DOACs in elderly patients remains to be fully defined. Phase III trials revealed that DOACs were at least as safe and effective as dose‐adjusted warfarin,^13^ however, elderly and frail patients were represented to a lesser extent. Recently, several real‐world studies have confirmed the safety and efficacy of DOACs in the elderly, although the frailty was not likely to be mentioned.^14^, ^15^ In the present study, bleeding events were significantly less frequent in the DOAC group than with the warfarin group, and the frequency of thromboembolic events did not differ significantly between the two groups. When comparing the DOAC and warfarin groups, the CrCl was significantly lower in the warfarin group than the DOAC group (37.2 ± 15.6 vs 46.9 ± 15.7, *P* < .01), and the usage of antiplatelet drugs was significantly more frequent in the warfarin group than the DOAC group (32.1% vs 19.9%, *P* = .024). Because the development of bleeding or thromboembolic events depends not only on the intensity of oral anticoagulant therapy but also on the patients clinical characteristics, we could not compare the outcomes directly for DOACs vs warfarin. However, a multivariate analysis using a Cox proportional hazard model adjusted by the risk factors of bleeding including the CrCl and usage of antiplatelet drugs revealed that warfarin therapy was associated with the development of bleeding events. Therefore, we are probably safe in thinking that DOACs can be used more safely than warfarin in nonsevere frail octogenarians. On the other hand, the bleeding patients receiving DOACs had a higher CFS score than the nonbleeding patients receiving DOACs (4.7 ± 0.8 vs 4.0 ± 0.9, *P* = .043), and all of the bleeding patients had a CFS score of ≥4. Hence, DOACs should be used carefully and with caution in patients with a high CFS score. The proposed algorithm for the management of frail patients also suggests that DOACs may be appropriate in nonsevere frail patients.^4^ However, it is important to note that the analysis included patients aged 65 years and older, and it has not been validated. We face in the daily clinical practice of geriatric patients, that there is a growing need for large trials in very old frail populations.

### 4.4 Antiplatelet therapy in combination with OACs

Biologic plausibility suggests that antiplatelet therapy in combination with OACs could lead to worse bleeding outcomes. However, a multivariate analysis using a Cox proportional hazard model revealed that antiplatelet therapy in combination with OACs was not associated with the development of bleeding events. Moreover, only 1 patient treated with DAPT in combination with OACs experienced bleeding events during the follow‐up period. A consensus has not been reached about the optimal duration of DAPT after PCI in AF patients treated with OACs. Moreover, DOACs may be used as an alternative to antiplatelet drugs in patients with stable CAD in the future.^16^ Following this, the duration of antiplatelet therapy varied among the patients treated with antiplatelet drugs in combination with OACs in the present study. The decisions regarding the OAC treatment during antiplatelet therapy were made by the each attending physician. Antiplatelet drug administration tended to be more likely to be stopped early because of the fear of bleeding. As a result, the antiplatelet therapy may not have been associated with the development of the bleeding. Further research is necessary to seek the optimal antiplatelet therapy in combination with OACs in this population.

### 4.5 Assessment of the prescription of inappropriate doses of OACs

Whether the dose recommendations of DOACs are adhered to in clinical practice remains a major concern, especially among elderly patients. In this study, approximately 30% of patients received inappropriate doses of DOACs according to the approved Japanese recommendations. As shown in a previous study, in which the majority of the patients on inappropriate doses of DOACs were found to be on a lower dose than recommended,^17^, ^18^ we also found that under‐treatment was more frequent than an over‐treatment in this setting. This is probably due to the fear of bleeding complications. Moreover, in the group with DOACs alone, given that an antidote was available only for dabigatran,^19^ prescribing lower doses makes clinicians more confident of a safer prescription. On the one hand, an inappropriate dose of warfarin was also often prescribed in the clinical practice even though an antidote was available for warfarin. The disadvantages of warfarin over DOACs are a variable dose regimen, it requires frequent drug monitoring, and it has interactions with some drugs and food.^20^ In general, elderly patients have multiple comorbid conditions that increase their risk of being exposed to polypharmacy. Besides, it is likely physically difficult for elderly patients with AF to go to regular INR checks at warfarin clinics even if their frailty is not so severe. This makes the elderly patients prone to difficulties in keeping up with variable warfarin dosages. These disadvantages may be derived from a poor medication adherence and thus an inappropriate dose. Actually, the present study showed that an inappropriate dose of warfarin was prescribed more often than inappropriate doses of DOACs (34.6% vs 23.1%, OR: 1.92, 95% CI: 1.12‐3.29, *P* = .043).

### 4.6 Safety and efficacy outcome according to the prescribed OACs and dosages

The incidence of bleeding and thromboembolic events did not differ among appropriate doses of DOACs, inappropriately low doses of DOACs, and inappropriately high doses of DOACs. Biologic plausibility suggests that lower doses of DOACs could lead to worse thromboembolic outcomes, and higher doses of DOACs could lead to worse bleeding outcomes. An analysis of the relation between DOAC dosages and clinical outcomes showed that inappropriately low doses of DOACs for ischemic stroke prevention in AF are related to worse clinical outcomes.^21^ However, the medication adherence was not discussed in the article. The importance of adherence to anticoagulants for ischemic stroke prevention in AF patients is already well established. In this study, follow‐up finished when patients discontinued the medication, so good adherence to DOACs was maintained during follow‐up periods. Our findings suggest that inappropriately low doses of DOACs may be a better alternative for prevention of thromboembolic events in elderly patients, as long as good adherence to DOACs is maintained. On the other hand, the follow‐up periods in patients treated with inappropriately high doses of DOACs were slightly shorter than that in those treated with appropriate doses of DOACs or inappropriately low doses of DOACs. This is probably due to the fear of bleeding in addition to the physicians awareness that the prescription high doses of DOACs are inappropriate. The low incidence ratio of bleeding events in patients treated with inappropriately high doses of DOACs could be related to their short follow‐up periods. Besides, the prescription of inappropriately high doses of DOACs was less frequent than that of appropriate doses of DOACs or inappropriately low doses of DOACs, which might have resulted in statistical bias. The individual assessment of the patient risks for bleeding and thromboembolic events often plays a major role in guiding the choice of DOACs and dosage, in particular in the elderly. While an appropriate dose prescription of DOACs is certainly mandatory, such appropriateness remains to be further elucidated. On the other hand, the safety and efficacy of warfarin are dependent on maintaining the INR value within the target range. This finding was supported in previous studies that had shown that inappropriate INR monitoring increases the risk of bleeding events.^22^, ^23^ Indeed, we observed that supratherapeutic INR values and bleeding events were more frequent in patients treated with an inappropriate dose of warfarin rather with an appropriate dose of warfarin. It seems that at least nonseverely frail octogenarians that have difficulty with warfarin dose adjustments should be considered for switching from warfarin to DOACs to avoid bleeding events.

### 4.7 Limitations

This study had some potential limitations. First, a single measure of frailty was used, so patients may have been misclassified based on other definitions of frailty. Second, this study was a retrospective and observational study conducted at a single center. The selections of DOACs or warfarin were not randomized as the decisions were made by the attending physicians. There were variations in the indication for DOACs or warfarin. The observation period also differed according to the OAC treatment regimen. Third, this study had a small number of patients, which might have resulted in a statistical bias. Especially, there were only 28 patients taking edoxaban when our survey was conducted (January 2011 to August 2017), because of the limited spread within the market during that early period after it obtained approval. Fourth, the follow‐up duration was relatively short. Further research is necessary with more patients and a long‐term follow‐up.

## 5 CONCLUSIONS

OACs are substantially safe and effective for reducing the risk of AF‐associated thromboembolic events in nonsevere frail patients over 80 years of age. Particularly, DOACs can be used more safely than warfarin.

## CONFLICT OF INTEREST

TI has received grant support through his institution from Daiichi Sankyo and Bayer Healthcare and honoraria for lectures from Bayer Healthcare, Bristol‐Myers Squibb, Pfizer, Daiichi Sankyo, and Ono Pharmaceutical. Regarding this study, all authors declare that there are no potential conflicts of interest.
